# Cost-Effectiveness of Lifestyle-Related Interventions for the Primary Prevention of Breast Cancer: A Rapid Review

**DOI:** 10.3389/fmed.2019.00325

**Published:** 2020-02-05

**Authors:** Martine Bellanger, Katharine Barry, Juwel Rana, Jean-Philippe Regnaux

**Affiliations:** ^1^MOS Research Unit, Department of Social Sciences, Ecole des Hautes Etudes en Sante Publique, Rennes, France; ^2^Institut de Cancerologie de l'Ouest, Nantes, France; ^3^International Breast Cancer and Nutrition Project, Lafayette, LA, United States; ^4^Department of Biostatistics and Epidemiology, University of Massachusetts Amherst, Amherst, MA, United States; ^5^Center CRESS - INSERM U1153, EpiAgeing Team, Paris, France

**Keywords:** breast cancer, primary prevention, cost-effectiveness, lifestyle, behavior

## Abstract

**Background:** In 2018, the global estimate of newly diagnosed breast cancer cases among women totaled 2.1 million. The economic and social burden that breast cancer places on societies has propelled research that analyzes the role of modifiable risk factors as the primary prevention methods. Healthy behavior changes, moderated alcohol intake, healthy body weight, and regular physical activity may decrease the risk of breast cancer among women. This review aimed to synthesize evidence on the cost-effectiveness of lifestyle-related interventions for the primary prevention of breast cancer in order to answer the question on whether implementing interventions focused on behavior changes are worth the value for money.

**Methods:** A rapid review was performed using search terms developed by the research team. The articles were retrieved from MEDLINE and the Tufts Medical Center Cost-Effectiveness Analysis Registry, with an additional web search in Google and Google Scholar. Comparisons were performed on the cost-effectiveness ratio per quality-adjusted life-year between the interventions using a league table, and the likelihood of cost-effective interventions for breast cancer primary prevention was analyzed.

**Results:** Six studies were selected. The median cost-effectiveness ratio (in 2018 USD) was $24,973, and 80% of the interventions had a ratio below the $50,000 threshold. The low-fat-diet program for postmenopausal women was cost-effective at a societal level, and the physical activity interventions, such as the Be Active Program in the UK, had the best cost saving results. A total of 11 of the 25 interventions ranked either as highly or very highly likely to be cost-effective for breast cancer primary preventions.

**Conclusion:** Although the review had some limitations due to using only a few studies, it showed evidence that diet-related and physical-activity-related interventions for the primary prevention of breast cancer were cost-effective. Many of the cost-effective interventions aimed to reduce the risk of non-communicable diseases alongside breast cancer.

## Introduction

Breast cancer has been ranked as the leading cause of cancer deaths in over 100 countries, accounting for 11.6% of all cancer deaths worldwide (1, 2). In 2018, 2.1 million women were newly diagnosed with breast cancer, and an estimated 626,679 women died due to breast cancer (2). Economically, breast cancer has been associated with increased healthcare costs and productivity losses (1–5). Among 27 European Union countries, breast cancer had the second largest share of overall cancer costs (12%), after lung cancer (15%) (€126 billion in 2009) (3). Low- and middle-income countries have experienced disproportionately high amounts of productivity loss, incidence, and mortality of women due to breast cancer (1, 3, 4). In 2012, breast cancer was found to contribute to the highest productivity loss among women in all but one BRICS countries (Brazil, Russia, India, China, and South Africa), representing 0.33% of their gross domestic product (4).

In recent years, the role of modifiable health behaviors in cancer prevention has been extensively studied (5–9). Associations were found between an increased risk in breast cancer and various lifestyle factors such as alcohol consumption, physical inactivity, exogenous hormone use, and excessive exposure to ionizing radiation (2). A research study which combined over 53 analyses on the links between alcohol and breast cancer onset found that with each increase of 10 g of daily alcohol consumption, women increased their risk for developing breast cancer by 7% (10). Over 100 studies which observed the association between weight and fat distribution and the development of breast cancer have found that women who are overweight or obese have 30–50% higher risk of developing postmenopausal breast cancer compared to women with a normal body mass index (BMI) (1, 5). An estimated 2.7 billion US dollars (USD) was spent on healthcare costs worldwide due to breast cancer that is attributed to physical inactivity (1, 3, 4).

To reduce the risk of breast cancer, primary prevention measures can focus on women who adopt healthy behaviors such as maintaining a normal weight, breastfeeding, minimizing alcohol consumption, eating a balanced diet, reducing stress, and decreasing the use of long-term hormone replacement therapy (11–14). Over 20 weight loss support programs have shown success in reducing the risk of breast cancer among postmenopausal participants by helping these women reach a normal BMI (8, 12).

The control of breast cancer through both early detection and primary prevention is of high priority in order to decrease the incidence and the premature mortality among women and to reduce the economic losses worldwide (11, 15). It is important to shed light on the benefits of investing in the primary prevention for breast cancer. Cost-effectiveness analysis can help in showing how to get the most of the available resources. A few published reviews on the cost-effectiveness of cancer interventions include the prevention strategies for breast cancer such as screening and chemoprevention, but lifestyle-related interventions were not included (16–19).

Our study aimed to review and synthesize the evidence on the cost-effectiveness of lifestyle-related interventions for the primary prevention of breast cancer. The objective of this review was to provide up-to-date evidence on the cost-effectiveness of the breast cancer prevention interventions focused on healthy weight programs, balanced diet interventions, physical activity (PA) programs, limited alcohol consumption interventions, and tobacco cessation programs. A rapid review approach, which aims to systematically synthesize the available evidence within a “limited time and resource framework,” was adopted to summarize the relevant information (20–23).

## Methods

### Rationale for a Rapid Review

Systematic reviews provide a rigorous and reproducible method to collect and summarize the available current evidence in the literature. They require very intensive resources and time to be conducted. They often fail to answer the research question when no or little relevant evidence is available. Rapid reviews have emerged as an alternative to address this issue. They are a novel form of systematic review which aim to produce faster and relevant evidence following the same methodological steps of a systematic review (24). They are useful to synthetize evidence for new or emerging research topics as well as to update previous reviews. Different approaches to conduct rapid reviews have been described (20–23). However, there is no recommendation on which shortcuts to use to conduct a rapid review faster than a systematic review. These may include: (1) more targeted research questions, (2) limited set of data sources searched, and (3) the use of only one reviewer for the study selection and/or the data extraction process. The finding synthesis is made of a descriptive/narrative summary instead of a qualitative summary plus meta-analysis (20–23).

### Protocol and Registration

A pre-specified review protocol was developed and followed for all of the methods (MB, JPR, and KB). The Preferred Reporting Items for Systematic Review (PRISMA) guidelines were used to report our findings (25).

### Information Sources and Search Strategy

The studies were identified using electronic databases. We searched MEDLINE via PubMed from its database inception until January 2019. A second database, the Tufts Medical Center Cost-Effectiveness Analysis Registry (www.cearegistry.org), was searched from 2014 to 2017 since a systematic review performed by Winn et al. summarized evidence on the cost-utility analysis of cancer prevention and treatment with studies dated up to 2013 (19). That systematic review was identified in the studies retrieved from the Medline search. We hand-searched reference lists from all of the studies and review articles included. Additional literature was searched using Google and Google Scholar.

The search terms were developed by the research team in collaboration with a faculty librarian. We used the following Population, Intervention, Comparison, Outcome (PICO) framework to identify the relevant terms: P: breast cancer, I: primary prevention, and O: cost-benefit analysis. The complete MEDLINE search strategy is presented in Supplementary Table 1. The search query was developed using index vocabulary (MESH) and free-text words. To test the search equation, we manually identified four relevant studies, and then based on the results of the testing search, we modified the final strategy to ensure that the relevant titles were included.

### Inclusion and Exclusion Criteria

To be included, the studies had to fulfill the PICO framework:

1. Populations: Adult women aged 16 years and older with no diagnosed breast cancer.
2. Interventions: Studies considering lifestyle-related primary prevention interventions such as dietary interventions, weight-loss-related interventions, PA interventions or physical exercise programs, alcohol consumption reduction interventions, and/or tobacco use reduction programs. The interventions were identified and informed based on international literature and previous studies (26–29). Studies related to early detection and diagnosis testing, chemoprevention (such as raloxifene or tamoxifen), surgical interventions (such as mastectomy), and ionizing radiation were excluded since the review focused on the lifestyle-related interventions. All interventions conducted on women diagnosed with breast cancer (i.e., tertiary prevention) were also excluded.
3. Comparators: Women without interventions, women with standard care or status quo, such as usual diet or current practice for PA, also called “usual care.”
4. Outcomes: The primary outcomes of the cost-effectiveness analysis were the costs and the quality-adjusted life-years (QALYs) or the disability-adjusted life-years (DALYs) and the incremental cost-effectiveness ratio (ICER) that considers the change in the costs and the effects of interventions on breast cancer, including other non-communicable diseases (NCDs) or not, compared to the status quo.
5. Study design: We applied no restriction on the type of study eligible for this review. We excluded any reports without results. We did not consider published letters or comments to be included.

Only the articles published in English were considered for this review.

### Selection of Sources of Evidence

All search results were imported and de-duplicated using Covidence Software (https://www.covidence.org). The title/abstracts and the full text were screened by two reviewers (JPR and MB). One reviewer (MB) screened all of the abstracts and the full text of the relevant references. A second reviewer (JPR) double-checked 15% (200/2,944) of the abstracts and inspected all of the full text of the rejected articles (185/191) to ensure that no relevant study was excluded. Disagreements were resolved after discussion.

### Data Items and Data Extraction Process

Two reviewers (MB JR) extracted data from the studies included. The data extraction form was piloted and modified as required based on the feedback from the team. The data were extracted from all of the studies included using a standardized template to capture optimal information. The extracted data about the general information of the published studies was collected in an EXCEL spreadsheet.

### Critical Appraisal of Individual Sources of Evidence

The quality of the selected studies was assessed (MB, JR, and JPR) using the guidelines recommended by Drummond and Jefferson for cost-effectiveness analysis studies (30). The quality of the study was determined by analyzing three categories: (1) study design, (2) data collection methods (e.g., model input such as outcome measures, cost components, and estimates), and (3) interpretation of results (e.g., time horizon, discount rates, sensitivity analysis, including probabilistic sensitivity analysis, and relevance of alternatives compared). To rate the quality of the evidence, we used a three-point scale for each item, as suggested in previous studies by Gerard et al. and Zelle and Baltussen. The final percentage ranges were thus expressed, and the overall quality of the study was set as in Zelle and Baltussen (31, 32). Lastly, review commentaries from the Center for Reviews and Dissemination (CRD) of the University of York were also used to match our quality assessment (https://www.crd.york.ac.uk/). Of note is the fact that since there is no standardized method to critically appraise the quality of the studies included in a systematic review, we considered the guidelines recommended in the health economic evaluation as the most appropriate for our rapid review.

### Synthesis of Results

We used a narrative synthesis to present the main findings of the studies and the different primary interventions selected. To compare the findings between studies, the non-USD cost-effectiveness ratios were converted into USD using the exchange rate factors for the price-year given in the studies. All ICERs were then inflated to 2018 USD based on the consumer price index from the Bureau of Labor Statistics (https://www.bls.gov/cpi/data.htm), as was done in previous studies (33). Median ICERs were estimated after inflation adjustment. A cost-effectiveness league table was constructed to present the ICER of the primary health interventions evaluated (34). The likelihood level of the cost-effectiveness of the intervention for breast cancer alone was estimated by extrapolating the incremental QALY required to get an ICER equal to $50,000, the most common WTP threshold used for the cost-effective strategies. Reductions in breast cancer incidence and breast cancer risk as well as the utilities associated with health states were analyzed. The interventions selected were those with high or very high likelihood levels of cost-effectiveness.

## Results

### Search Strategy and Study Identification

**Figure 1:**
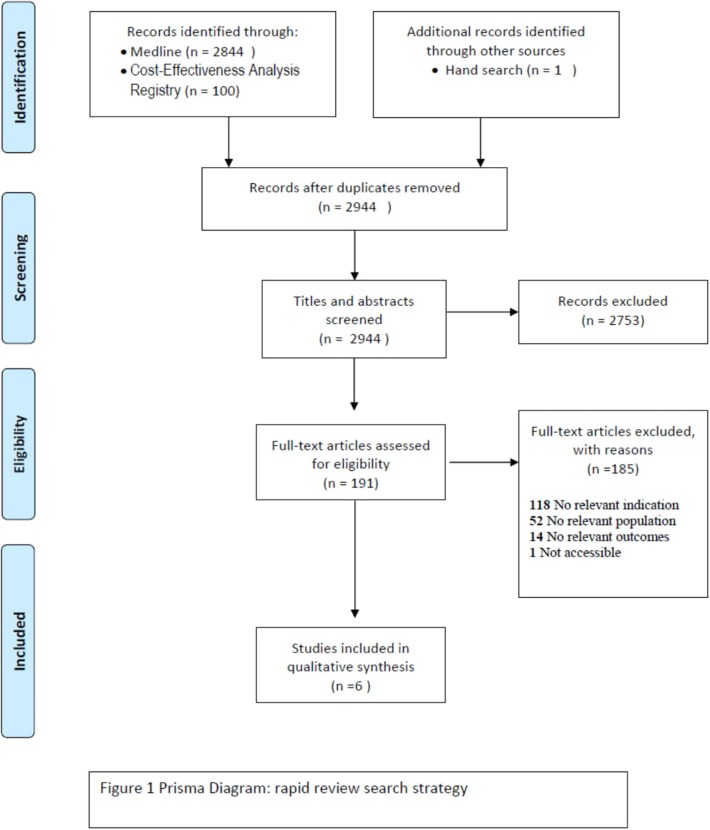
PRISMA.

The first step of the literature search for the primary prevention of breast cancer identified 2,955 references according to the outlined criteria above (Figure 1). The screening of titles and abstracts left 191 full texts to be examined. Further selection resulted in the exclusion of 185 studies that were ineligible for different reasons, such as irrelevant indication to our research question (*n* = 118), irrelevant population (*n* = 52), and irrelevant outcome measure (*n* = 14). One full text was not accessible. Six studies were considered for the qualitative analysis. Also, we found one protocol which analyzes the impact and the cost-effectiveness of the lifestyle interventions for breast cancer, but the results of this study will not be published until the end of 2019 (35).

### Characteristics of the Studies Included

The six studies included were published between 2007 and 2014. All of the studies were conducted in high-income countries (HICs): two studies were from the USA, and one study each was from Australia, Belgium, Netherlands, and UK (Supplementary Table 2). Two types of primary prevention-related interventions were evaluated: PA (*n* = 5) and diet (*n* = 2) (36–41).

Breast cancer was the primary focus of prevention, along with ovarian cancer only, in Bós et al. who analyzed the cost-effectiveness of a low-fat diet on these two cancers (37). In five of the studies, breast cancer was among other non-communicable diseases (NCDs), such as coronary heart disease, diabetes, stroke, and colorectal cancer, targeted by the primary prevention interventions, and it was included in the cost-effectiveness model (Supplementary Table 2).

All of the PA-related studies were carried out in a community setting, except for one study which combined PA and diet in a secondary care setting. There were three types of study designs: hypothetical cohorts, closed cohorts of a given population, and randomized control trials (RCTs). The adult populations with ages from 16 to 30, as well as the populations aged 50 and above, were the most commonly targeted groups (36, 38, 39, 41). However, menopausal women were targeted for the primary prevention of breast and ovarian cancer (Supplementary Table 2) (37, 40). The PA strategies compared no intervention or “usual care” to one or up to six strategies in one study (41). The inter-strategy comparison was made by Peels et al. (40).

All studies were either cost-effectiveness analyses (*n* = 5) or cost-utility analyses (*n* = 1) based on Markov models (*n* = 6). The model inputs (i.e., outcomes, utility values, and costs) were derived from RCTs (*n* = 3), from literature (*n* = 4), and from national databases (*n* = 3). A natural experiment was used in Frew et al. (39) (Supplementary Table 2).

In all studies, the reported costs and benefits were combined in an ICER (*n* = 5) or an incremental cost per utility ratio (ICUR) (*n* = 1). The additional costs per QALY gained were estimated in most studies. Only (38) estimated the ICER per DALY for the diet and exercise interventions. Final estimates were available in the country currency and price-year (*n* = 5). The time horizon used in the studies varied from 5 years to the lifetime horizon of the population studied. Different time horizons were used in the sensitivity analysis. In all studies but one, the cost-effectiveness analysis was presented from the perspective of the society, and in half of the studies, both the society and the healthcare payer perspectives were included. Society WTP thresholds are presented (Supplementary Table 2).

### Study Quality

Table 1 presents the quality of the six studies included, ranging from 74 to 89%. Bós et al. ranked the highest score for very good quality, followed by Frew et al. and Peels et al. (37, 39, 40), while the lowest score was found for Annemans et al. (36). All studies underperformed in category 2 (“data collection”). For instance, information on some model parameter sources was insufficient or not easily accessible, and total resource estimates were not reported separately from their unit costs and quantities for indirect costs. For domain 3 (“result analysis and interpretation”), the full score was not reached, mostly due to insufficient relevant alternative comparisons, except in Peels et al. (40). The price-year was not available only in one study, which hampers any inflation-adjusted estimation and comparison with the other interventions (36).

**Table 1:** Summary of quality assessment in percentage range^a^.

**References**	**Study design (14 points): research question, form of economic evaluation**	**Data collection (28 points): outcomes, costs, model, currency, and price**	**Result analysis and interpretation (26 points): time horizon, discount rate, sensitivity analysis, conclusions**	**Overall quality score**	**Final qualitative assessment**^**b**^
Annemans et al. (36)	100	68–73	81–88	74–78	Good
Foster et al. (38)	100	68–73	88–96	82–88	Good
Roux et al. (41)	100	68–73	88–96	82–88	Good
Frew et al. (39)	100	68–73	92–100	84–89	Very good
Peels et al. (40)	100	68–73	92–100	84–89	Very good
Bós et al. (37)	100	54–58	92–100	84–89	Very good

a *The score was reduced with two points when a non-appropriate item in a domain was observed as done by Zelle and Balthussen (32)*.

b *Final quality scoring adapted from Zelle and Balthussen as “poor quality (scoring 40–55%), good quality (scoring 55–70%), very good quality (scoring 71–85%), and excellent quality (scoring 86% or higher)” (32). The lowest bound of the score range gives the final quality level*.

Lastly, our quality assessment for the four studies published between 2007 and 2011 fit the assessment published by the CRD from the National Institute for Health Research. For the two studies published in 2014, our assessment fit the expected findings based on the available positive pre-review.

### Cost-Effectiveness Findings

The median cost-effectiveness (in 2018 USD) reported in the four studies, of which ICER/QALY was estimated and for which the price-year was available, was $24,973 (37, 39–41). From a societal perspective, 80% of the interventions had a ratio below $50,000 WTP threshold (as shown in Table 2). When the distribution across all of the interventions was assessed (i.e., including healthcare payer and society perspectives), 75% of the cost-effective ratios were below $50,000, 18% were between $50,000 and $100,000, and 7% were above $100,000.

**Table 2:** League table of incremental cost-effectiveness ratio by intervention, from a societal perspective and extrapolated likelihood of cost-effectiveness level for breast cancer (BC) for four studies included.

**References**	**Intervention type and comparator**	**2018 US$/QALY**	**Likelihood cost-effectiveness level for BC**
Frew et al. (39)	Base case analysis Be Active vs. no scheme, 5-years time horizon	721	Very high
Frew et al. (39)	Be active vs. no scheme, 2-years time horizon	3,374	Very high
Frew et al. (39)	Reduction physical activity over time Be Active vs. no scheme	3,850	Very high
Peels et al. (40)	Computer-tailored PA intervention: basic printed vs. usual care, lifetime horizon	11,606	Very high
Bós et al. (37)	Low-fat-diet-intervention women with high risk of breast cancer with fat intake ≥32% vs. usual diet, starting at age 50 years; lifetime horizon	12,600	Very high
Bós et al. (37)	Low-fat-dieta-intervention women with high fat intake at baseline >36.8% vs. usual diet, starting at age 50 years; lifetime horizon	15,468	High
Peels et al. (40)	Computer-tailored PA intervention: web-based basic vs. usual care, lifetime horizon	15,629	High
Roux et al. (41)	An 8-weeks community intervention for walking/NO; lifetime horizon	19,475	High
Bós et al. (37)	Low-fat-diet-intervention women with high risk of breast cancer with fat intake ≥32% vs. usual diet, starting at age 55 years; lifetime horizon	17,752	High
Bós et al. (37)	Low-fat-diet-intervention women with high fat intake at baseline >36.8% vs. usual diet, starting at age 55 years; lifetime horizon	18,583	High
Bós et al. (37)	Low-fat-diet-intervention women with high risk of breast cancer with fat intake ≥32% vs. usual diet, starting at age 60 years; lifetime horizon	18,647	High
Bós et al. (37)	Low-fat-diet-intervention women with high fat intake at baseline >36.8% vs. usual diet, starting at age 60 years; lifetime horizon	23,911	Medium high
Bós et al. (37)	Low-fat-diet-intervention women with high with high risk of breast cancer with fat intake ≥32% vs. usual diet, starting at age 65 years; lifetime horizon	24,451	Medium high
Roux et al. (41)	Exposure to an environment favoring a more active lifestyle/NO; lifetime horizon	34,827	Medium
Bós et al. (37)	Low-fat-diet-intervention women with high fat intake at baseline >36.8% vs. usual diet, starting at age 65 years; lifetime horizon	31,443	Medim low
Roux et al. (41)	Initial training session for walking program/NO; lifetime horizon	37,315	Medium low
Peels et al. (40)	Computer-tailored PA intervention: web-based environment vs. printed; 5-years time horizon	31,723	Medium low
Roux et al. (41)	Personal trainer intervention and financial incentives for PA/NO; lifetime horizon	40,657	Medium low
Bós et al. (37)	Low-fat-diet-intervention women with high risk of breast cancer with fat intake ≥32% vs. usual diet, starting at age 70 years; lifetime horizon	41,168	Low
Roux et al. (41)	Organized walking groups, social events for promoting PA/N; lifetime horizon	54,105	Very low
Peels et al. (40)	Computer-tailored PA intervention: printed environment vs. basic, 5-years time horizon	45,959	Very low
Bós et al. (37)	Low-fat-diet-intervention women with high fat intake at baseline >36.8% vs. usual diet, starting at age 70 years; lifetime horizon	51,197	Very low
Peels et al. (40)	Computer-tailored PA intervention: vs. basic web-based; 5-years time horizon	49,967	Very low
Roux et al. (41)	Intensive lifestyle modification program, for high risk diabetes 2 adults/NO; lifetime horizon	63,953	Very low
Roux et al. (41)	A 6-years community health education intervention (Stanford 5 City Project) vs. no intervention (/NO); lifetime horizon	93,457	Null

*ICER values or value ranges were ≤ 12,499 for very high likelihood, 12,500–17,499 for high, 17,500–22,499 for medium high, 22,500–27,499 for medium, 27,500–32,499 for medium low, 32,500–37,499 for low, 37,500–50,000 for very low and null for ICER > 50,000. The study of Annemans et al. (36) is not included since no price-year was available, and Foster et al. (38) was not included since ICER/DALY was estimated. In Bós et al. estimates are presented from intervention start; estimates from the start of randomization as well as ICERs for the payer perspective were available in the publication, but not presented here, for purpose of comparison with the other three studies (37). Source: league table adapted from Greenberg et al. and likelihood extrapolation made by co-authors of the review (34)*.

The low-fat-diet program for postmenopausal women, which is the sole study focusing only on breast cancer and ovarian cancer, was cost-effective from a societal perspective (37). When looking at the age of the program start, women who enrolled at age 70 vs. age 50 with a high fat intake at baseline and a high risk of breast cancer had over three times higher cost-effectiveness ratio.

PA interventions targeting five major NCDs, including breast cancer, were ranked first in terms of their cost-effectiveness (39). Specifically, the Be Active Program in the UK had the best value for money or was cost-saving (39). The computer-tailored PA interventions implemented in Netherlands, as well as some community-based PA in the US, were also among the most cost-effective (Table 2) (40, 41).

A total of 11 out of 25 interventions were assessed as likely to be cost-effective for the primary prevention of breast cancer, and their likelihood levels of cost-effectiveness were ranked as very high or high (Table 2). The incremental QALYs required for the current incremental costs of the intervention related to breast and ovarian cancer to make the ICER at $50,000 were three to five times lower than the actual incremental QALYs (37). The same order of magnitude was found in Roux et al. and Peels et al. (40, 41). In the study of Frew et al., the “Be Active” program was shown to produce societal positive net benefit and also exhibited the highest chance for the PA program to be deemed cost-effective for breast cancer (39) (Supplementary Table 3).

## Discussion

### Main Findings

This rapid review shows evidence of the cost-effectiveness of the diet-related interventions on breast cancer and ovarian cancer as well as the PA-related programs on breast cancer and other major NCDs. Our review also included interventions that addressed breast cancer alongside other NCDs, such as coronary heart disease, stroke, diabetes, and colorectal cancer. Only one study differed from that approach, focusing only on two gynecological cancers (37). The benefits and value of primary prevention interventions in reducing the disease risk other than cancer and improving the overall quality of life have been documented (36, 38–41). The cost-effectiveness ratio for all of the studies included was estimated by calculating the overall cost-effectiveness of these multi-factorial interventions.

Estimating the cost-effectiveness of the lifestyle-related interventions only for breast cancer vs. the cost-effectiveness of these interventions for all NCDs would likely result in higher ICERs since, for the same change in costs, the differences in QALYs for breast cancer alone, in the denominator of the ICER, might be smaller. However, the favorable cost-effectiveness ratios of diet and PA-related interventions for all NCDs would remain below $50,000 per QALY for breast cancer alone. Despite our communication with the authors of these studies, we were not able to get the ICERs for breast cancer alone. For the low-fat-diet interventions, based on personal communication from Bós, favorable ICERs were found for breast cancer alone, and all were below the $50,000 threshold (37). The primary prevention strategies assessed in this analysis were congruent with other well-accepted public health strategies published in 2016 (19). These well-accepted interventions had a median cost-effectiveness ratio of $48,000 in 2014, which solely focused on drug therapy and mastectomy for breast cancer prevention. Some experts considered these therapies to be cost-effective, and societies incorporated them as one of the main strategies for breast cancer prevention (19, 33, 40).

The long-term effects of PA interventions have been shown to make the primary prevention interventions cost-effective, which is very sensitive to the time horizon in the economic evaluation. The longer the time, the lower the cost-effectiveness ratio will be. Time is needed to observe the potential outcomes of a primary prevention. Overall, the benefits would be greater in the long term than in the short term. Of the seven interventions assessed in the USA by Roux et al., six of them were cost-effective over a 40-years time horizon (41). Some interventions would be unlikely to be cost-effective due to the short time horizon of 10 years. For instance, the cost-effectiveness ratio for the walking education program would increase from $27,000 per QALY to $147,000 per QALY (41). Peels et al. showed that the computer-tailored PA interventions, with advice three times over 4 months and targeting Dutch community-dwelling adults, achieved cost-effectiveness on a long time horizon (40). ICERs below the $27,800 WTP threshold were used for prevention interventions in The Netherlands. On a 5-years horizon, only the web-based tailored intervention was borderline cost-effective. The impacts of primary prevention may take years to be noticeable. Hence, investment in primary prevention programs may be limited due to the decision-makers' desire for higher impacts in a shorter time frame (42, 43).

To our knowledge, this rapid review is the first review of its kind that focused on the lifestyle prevention interventions such as healthy weight programs, nutrition and balanced diet interventions, PA programs, limited alcohol consumption interventions, and tobacco cessation programs, excluding a previous study based on breast cancer preventions that found limited evidence of the effectiveness of primary prevention interventions (40). A benefit of performing a rapid review was that such evidence of the cost-effective interventions on breast cancer, for which limited research is available, might have not been possible to be synthesized from a traditional systematic review. Despite the observations and recommendations over the last two decades, few cost-effectiveness analyses have targeted healthy people, although some evidences are available for breast cancer (19, 33). Winn et al. showed in their systematic review on the “cost-utility analysis of cancer prevention, treatment, and control” that breast cancer was ranked first in terms of cost-utility-analysis-related studies (29% of all studies in the review) (19). However, tertiary prevention (treatment) and secondary prevention represented the majority of all studies (i.e., 77 and 15%, respectively), while the remainder (8%) was for primary prevention. Within the primary prevention interventions of breast cancer, the majority of studies focused on chemoprevention therapy and mastectomy procedures (88%). Based on current publications, the study shared the same conclusion that “researchers have devoted relatively little attention” to the cost-effectiveness of primary prevention (33). In contrast, an estimated 40% of cancers could be prevented if time and resources were invested to identify the protective factors which individuals can take to avoid the onset of cancer (8, 12, 44). Moreover, several studies on NCDs including breast cancer and their lifestyle-related risk factors, such as physical inactivity and excess weight, recommended conducting cost-effectiveness analyses of these interventions (45–48).

### Limitations

Our study had several limitations. Firstly, the number of studies that could be included was limited. Only two types of interventions were identified: physical activity (in five studies) and diet (in two studies). The small number of interventions did not permit the differentiation of the primary-prevention-related impact of intervention on breast cancer. More studies might be required to reach such an impact of public health interventions. The lack of sufficient evidence on the primary prevention interventions in reducing breast cancer might hinder the economic evaluations of lifestyle-related interventions. Also, it might be a result of our rapid review strategy and the limited number of databases searched. However, similar limitations were observed in previous systematic reviews in a number of studies retrieved (19). Secondly, the review included some studies in which the interventions were targeted not only for breast cancer but also for other NCDs. This may limit the implications of our findings. However, we believe that the inclusion of those NCDs still made our findings comprehensive and inclusive for lifestyle-related interventions for breast cancer that could not have been selected otherwise. Thirdly, the study quality assessment of the breast cancer primary-prevention-related cost-effectiveness rapid review had some limitations. The specific challenges of public health economic modeling require particular attention, notably related to uncertainty, which we checked in the quality assessment of the studies selected. However, additional items required to be assessed especially when different study designs are used. Natural experiment studies increasingly used in the evaluation of public health interventions may provide high “real-world setting” relevance and higher external validity than the RCTs at the expense of internal validity, unless the authors of the study select the optimal control group. Additionally, the authors' conflicts of interest were omitted from the quality assessment. This might have resulted in a “publication bias” as observed in a previous systematic review (34). Including those items in the quality assessment grid in future systematic reviews will improve the comparison between the interventions.

There are further limitations. While physical inactivity, excess weight, and unhealthy diet are significant threats to worldwide populations, our cost-effectiveness estimates were limited to HICs only (15, 47, 48). Thus, it is difficult to extrapolate or generalize the findings of the study to other countries and settings. Finally, the policy interventions related to lifestyle behaviors were not included in our study, which might hamper some complementary health benefits of selected taxation policies (49–51).

## Conclusions

The rapid review of the six primary prevention studies highlighted that the use of PA programs and low-fat-diet interventions among particular subgroups of women had high cost-effectiveness. Many of the cost-effective interventions aimed to reduce the risk of NCDs alongside breast cancer, allowing public health professionals to use a holistic program addressing multiple aspects of a woman's health. Societies have invested in primary prevention drug therapies and surgical procedures for breast cancer, and the same investment can be made in the lifestyle interventions targeting breast cancer. We intend that a future systematic review will help in identifying the additional cost-effectiveness of lifestyle-related primary prevention of breast cancer.

## Author Contributions

MB, J-PR, and KB contributed to conceptualization and design. MB and JR collected and assembled information. All authors contributed to data analysis and interpretation, contributed to manuscript writing, and agree to be accountable of all aspects of the work.

### Conflict of Interest

The authors declare that the research was conducted in the absence of any commercial or financial relationships that could be construed as a potential conflict of interest.
