# Supplementary material for: Cost-Effectiveness of Lifestyle-Related Interventions for the Primary Prevention of Breast Cancer: A Rapid Review
Source: Front Med (Lausanne). 2020 Feb 5;6:325. doi: 10.3389/fmed.2019.00325 (PMC7013088; doi:10.3389/fmed.2019.00325)
Supplement: Supplementary file 1 [file Table_1.pdf]

Supplementary Material

Supplementary Table 1 PubMed Search Strategy for primary prevention of breast cancer (2019 January)

Concept	Search terms
Population	1. Breast neoplasms [MeSH Terms]<br>2. Breast Neoplasm*[Title/Abstract]) OR Breast Cancer [Title/Abstract] OR Breast Tumor* [Title/Abstract] OR Breast Carcinom*[Title/Abstract] OR Breast (Breast* n3 neoplasm*[Title/Abstract] OR Cancer prevention[Title/Abstract])<br>3. 1 OR 2
Intervention	4. Primary Prevention [MeSH Terms]<br>5. Diet [MeSH Terms]<br>6. nutrition therapy [MeSH Terms]<br>7. Diet, Reducing [MeSH Terms]<br>8. Exercise [MeSH Terms]<br>9. Physical Exertion [MeSH Terms]<br>10. Drinking [MeSH Terms]

	11. Drinking Behavior[MeSH Terms]<br><br>12. Tobacco Smoking[MeSH Terms]<br><br>13. Primary prevention[Title/Abstract] OR Primary preventive [Title/Abstract] OR Prevention [Title/Abstract] OR education*[Title/Abstract] OR multifactorial [Title/Abstract] OR behavioral change [Title/Abstract] OR health promotion [Title/Abstract] OR counselling [Title/Abstract] OR diet*[Title/Abstract] OR nutrition*[Title/Abstract] OR exercise*[Title/Abstract] OR physical activity [Title/Abstract] OR alcohol [Title/Abstract] OR consumption [Title/Abstract] OR food [Title/Abstract] OR tobacco [Title/Abstract] OR lifestyle [Title/Abstract])<br><br>14. 4 OR 5 OR 6 OR 7 OR 8 OR 9 OR 10 OR 11 OR 12 OR13
Outcome	15. Cost benefit analysis [MeSH Terms]<br><br>16. Economics [MeSH Terms]<br><br>17. Economic evaluation* [Title/Abstract] OR Cost-utility [Title/Abstract] OR Cost benefit [Title/Abstract] OR Cost analysis [Title/Abstract] OR Cost-effectiveness [Title/Abstract] OR Health evaluation [Title/Abstract] OR Cost* [Title/Abstract] OR Prices[Title/Abstract] OR pricing [Title/Abstract] OR Economic* [Title/Abstract] OR Qaly* [Title/Abstract] OR daly* [Title/Abstract] OR Value for money[Title/Abstract] OR Return on investment[Title/Abstract])<br><br>18. 15 OR 16 OR 17
Results	19. 3 AND 14 AND 18

1. Breast neoplasms[MeSH Terms]
2. Breast Neoplasms[Title/Abstract]) OR (Breast Cancer[Title/Abstract] OR Breast Tumor[Title/Abstract] OR Breast Tumors[Title/Abstract] OR Breast Carcinoma[Title/Abstract] OR Breast Carcinomas[Title/Abstract])) OR (Breast$ adj3 neoplasm$[Title/Abstract] OR Cancer prevention[Title/Abstract])
3. 1 OR 2 **(356705)**
4. Primary Prevention [MeSH Terms]
5. Diet [MeSH Terms]
6. nutrition therapy [MeSH Terms]
7. Diet, Reducing [MeSH Terms]
8. Exercise [MeSH Terms]
9. Physical Exertion [MeSH Terms]
10. Drinking [MeSH Terms]
11. Drinking Behavior[MeSH Terms]
12. Tobacco Smoking[MeSH Terms]
13. Primary prevention[Title/Abstract] OR Primary preventive[Title/Abstract] OR Prevention[Title/Abstract] OR education$[Title/Abstract] OR multifactorial[Title/Abstract] OR behavioral change[Title/Abstract] OR health promotion[Title/Abstract] OR counselling [Title/Abstract] OR diet$[Title/Abstract] OR nutrition$[Title/Abstract] OR exercis$[Title/Abstract] OR physical activity[Title/Abstract] OR alcohol[Title/Abstract] OR consumption[Title/Abstract] OR food[Title/Abstract] OR tobacco[Title/Abstract] OR lifestyle[Title/Abstract])
14. 4 or 5 or 6 or 7 or 8 or 9 or 10 or 11 or 12 or 13 **(2842133)**
15. Cost benefit analysis [MeSH Terms]
16. Economics[MeSH Terms]
17. Economic evaluation$[Title/Abstract] OR Cost-utility[Title/Abstract] OR Cost benefit [Title/Abstract] OR Cost analysis[Title/Abstract] OR Cost-effectiveness[Title/Abstract] OR Health evaluation[Title/Abstract] OR Cost$[Title/Abstract] OR Prices[Title/Abstract] OR pricing[Title/Abstract] OR Economic$[Title/Abstract] OR Qualy[Title/Abstract] OR daly[Title/Abstract] OR Value for money[Title/Abstract] OR Return on investment[Title/Abstract])
18. 15 or 16 or 17 (**965425)**
19. 3 AND 14 AND 18 **(2844)**
