# Supplementary material for: Cost-Effectiveness of Lifestyle-Related Interventions for the Primary Prevention of Breast Cancer: A Rapid Review
Source: Front Med (Lausanne). 2020 Feb 5;6:325. doi: 10.3389/fmed.2019.00325 (PMC7013088; doi:10.3389/fmed.2019.00325)
Supplement: Supplementary file 2 [file Table_2.pdf]

Supplementary Material

Supplementary Table 2 Overall findings of the studies reviewed in terms of cost-effectiveness of primary prevention interventions

**Author, Year, Country**	**Population and Setting**	**Intervention**	**Comparator/ reference intervention**	**Type of Economic evaluation & Data sources**	**Perspective of the study**	**Price year, time horizon discount rate**	**Authors 'conclusions Cost / QALY, Cost/DALY**
Annemans et al 2007, Belgium	Community setting -fitness centre Hypothetical cohort of 3 target populations (**1**)(**2**)(**3**)	Controlled and maintained **physical exercise**	Do nothing (current practice or "usual care")	Cost-Utility Analysis (CUA) Six state transition Markov model (1) to (6), (1)healthy, (2) coronary heart disease (CHD), and (6) **breast cancer.** Model inputs from literature	Public payer Society perspective Productivity loss associated with absenteeism included	No price year 25-year time horizon 3% discount rate (benefits and costs)	Incremental cost-utility ratio (ICUR) range based on public subsidy (0 or €500) Cost-effective interventions, effects on several diseases, (uncertainty,probalisitic sensitivity analysis)
	(**1**)30 years, old, body mass index (BMI) 26, cholesterol 190, systolic blood pressure (SBP) 120	For cohort (**1**)					Dominant to cost-effective
	(**2**) 40 years, old, BMI 30 cholesterol 210, SBP 130	For cohort (**2**)					Dominant to cost-effective
	(**3**)50 years, old, BMI 32 cholesterol 250, SBP 140	For cohort (**3**)					Dominant to cost-effective
Foster et al (2011), Australia	Secondary care setting. Target population: overweight or obese Australian adults aged 20 and above in 2003, assumed to be recruited via mass media campaigns and postal mailing	**Diet** and **exercise**		Cost-Effectiveness Analysis (CEA), Multistate life table Markov model Among the nine obesity-related diseases, **breast cancer** was included Model inputs from RCT & national database	Health sector Travel costs & Patient time	AU dollars (AUD) Price year 2003 100-year time horizon 3% discount rate (benefits and costs)	ICER range based on no inclusion and inclusion of travel costs and patient time, sensitivity analysis ICER under AUD 50,000, only with health care costs for DASH. For diet alone, under for both But only 0.1% obesity burden averted
	.	Dietary Approach to Stop Hypertension (DASH) including fruits & vegetables & and low fat and sugar and exercise program. Meeting with dieticians & exercise physiologists	No intervention				Cost-effective to dominated
		A diet alone: education program of monthly meetings with dieticians.	No intervention				Cost-effective

Supplementary Table 2 continued

Author, Year, Country	Population and Setting	Intervention	Comparator/ reference intervention	Type of Economic evaluation & Data sources	Perspective of the study	Price year, time horizon, discount rate	Authors 'conclusions Cost / QALY, Cost/DALY
Roux et al (2008), USA	Community setting, various locations for different physical activity (PA). Adult population aged 25 to 64 years, in 2004, free of disease associated with physical inactivity	Five **physical activity (PA)** interventions		CEA, Flexible state transition Markov model simulation of close cohort (CHD, ischemic stroke, type 2 diabetes, **breast** & colorectal **cancer.** Model inputs from literature & national database	Society perspective Patient time included WTP threshold:$50,000	Price year 2003 40-year time horizon 3% discount rate (costs)	Cost-effective Community intervention strategies for PA ICER sensitive to time horizon, the shorter the less cost-effective the intervention (probabilistic sensitivity analysis)
		A six-year community health education intervention (Stanford 5 City Project)	No intervention				Cost-effective (50% chance below $50,000)
		An eight-week community intervention for walking (Wheeling Walks)	No intervention				Cost-effective
		Organized walking groups, social events for promoting PA	No intervention				Cost-effective
		Initial training session for walking program	No intervention				Cost-effective
		Personal trainer intervention, & financial incentives for PA	No intervention				Cost-effective
		Intensive lifestyle modification program, for high risk diabetes 2 adults	No intervention				Cost-effective
		Exposure to an environment favoring a more active lifestyle.	No intervention				Cost-effective
Frew et al (2014), UK	Local city-run leisure centers for city-dwelling adults in Birmingham aged 16 to 70 years, as closed cohort for simulation. 707 new participants aged 16 and over from nineteen council centers in the Be Active program, June to August 2010	For **Be Active** participants of the scheme: free access to fitness gyms (induction session included), swimming pools, and group fitness classes at certain times of days and weekends.	No intervention: "usual care: paying for access to leisure centers" For PA before program, data from the new enrolled Be Active participants	CEA Markov model simulation of close cohort of the Birmingham adults, all healthy at start. **Breast cancer** among 5 diseases. Model input from natural experiment and from literature review	Health care perspective Society perspective	Price year 2009/2010 5 year and 2 to 10-year time horizon for sensitivity analysis 3.5% discount rate (benefits & costs)	ICER range based on time horizon 10 to 2 years Cost-effective program (sensitivity analysis) Cost (£) /QALY range Strongly dominant to Cost-effective
				Cost-Benefit Analysis (CBA): Willingness to Pay (WTP)			Annual net benefit to society

Supplementary Table 2 continued

Author, Year, Country	Population and Setting	Intervention	Comparator/ reference intervention	Type of Economic evaluation & Data sources	Perspective of the study	Price year, time horizon discount rate	Authors 'conclusions Cost / QALY, Cost/DALY
Peels et al (2014), Netherlands	Communities of the Municipal Health Council regions participating in the study (2010-2011), same characteristics (i.e. urbanity, percentage of low and high socioeconomic status, and percentage of migrants) with participants assigned to one of the five interventions (RCT) 1,729 people aged over fifty recruited via direct mailing	Computer -tailored intervention **physical activity (PA**) intervention: tailored advice delivered to participants 3 times over four-month period, in four different versions, printed or web-based and with environment information (e.g. cycling routes, home exercises) or without (so-called basic)	Care as usual Web-based versus printed Environment versus basic	CEA, Chronic Disease Model: Markov state-transition model with several PA related chroni (CHD, stroke, diabetes, **breast cancer** & colon Cancer. Model inputs from the RCT and national database	Society perspective Threshold WTP€20,000	Price year 2011 5 year time horizon 4% discount rate for costs and 1.5% for benefits (0 used in the sensitivity analysis	Preferred intervention: on long time horizon, printed intervention on a 5 year horizon, web-based intervention Influence of time horizon 95% IC Probabilistic sensitivity analysis
		Basic printed	Care as usual				Cost-effective over lifetime and 5 years
		Printed environment	Basic printed				Dominated
		Web-based basic	Care as usual				Cost-effective over lifetime & 5 years
		Web-based environment	Printed				Not cost-effective over 5 years
		Web-based environment	Basic web-based				Dominated
Bos et al (2011), USA	Primary care setting: US Clinical centers for RCT 48,835 postmenopausal women aged 50 to 79 years and without prior cancer Eight years follow up. With two subgroups (1) (2)	Dietary intervention: dietary modification program Women health initiative Randomized Control Dietary Modification (VHI-DM).		CEA, Markov cohort model. **Breas**t **cancer** and ovarian cancer with five age cohorts (50y, 55y, 60y, 65y, 70y). Model inputs from literature & RCT	Society perspective Health care payers (public and private) Opportunity costs included Threshold WTP€50,000	Price year 2008 Lifetime horizon 3% discount rate	Cost-effective strategies to prevent **breast** and ovarian **cancer from the societal perspective** 95% IC below acceptable US threshold, sensitivity analysis
	(1) women with high fat intake at baseline >36.8%	Low-fat dietary intervention	Usual diet		Society perspective		Cost-effective for all age start (50,55,60,65,70)
					Health care payers		Not cost-effective for private payers (age 50,55), cost-effective for Medicare (65,70)
	(2) women with high risk of breast cancer with fat intake ≥32%	Low-fat dietary intervention	Usual diet		Society perspective		Cost-effective for all age start (50,55,60,65,70)
					Health care payers		Not cost-effective for private payers (age 50,55) cost-effective for Medicare (65,70)
