# Supplementary material for: Cost-Effectiveness of Lifestyle-Related Interventions for the Primary Prevention of Breast Cancer: A Rapid Review
Source: Front Med (Lausanne). 2020 Feb 5;6:325. doi: 10.3389/fmed.2019.00325 (PMC7013088; doi:10.3389/fmed.2019.00325)
Supplement: Supplementary file 3 [file Table_3.pdf]

Supplementary Material

Supplementary Table 3 Estimated Incremental QALY required if the current Incremental cost were based on a $50000 WTP threshold

		Incremental cost	USD	current Incremental QALY	Required incremental QALY for cost effective	Disease risk decrease with PA
Freew et al 2014	Basce case analysis (5 years) Be active versus no scheme	24	38	0,06	0,00076	0.004(0.003,0.005)
	Time horizon 2 years, be active versus no scheme	92	146	0,05	0,00292	
	Reduction physical activity over time be active versus no scheme	63	100	0,03	0,00126	
						Cumulative Hasard ratio (HH) Year 8
Bos et al 2011	Low fat dietary intervention women with high with high risk of breast cancer with fat intake ≥32%, versus usual diet, start age 50y	1003	1003	0,095	0,02016**	0,778 (p<0.01)
	Low fat dietary intervention women with high fat intake at baseline >36.8% versus usual diet, start age 50y	1111	1111	0,086	0,0222**	
	Low fat dietary intervention women with high with high risk of breast cancer with fat intake ≥32%, versus usual diet, start age 55y	991	991	0,083	0,01982**	
	Low fat dietary intervention women with high fat intake at baseline >36.8% versus usual diet, start age 55y	1160	1160	0,075	0,0232**	
	Low fat dietary intervention women with high with high risk of breast cancer with fat intake ≥32%, versus usual diet, start age 60y	1100	1100	0,07	0,022**	
						BC incidence decrease
Roux et al 2008	An eight-week community intervention for walking (Wheeling Walks) / NO	700	700	0,049	0,014	15 to 58 per 100,000
						BC incidence decrease
Peels et al 2014	Computer -tailored PA intervention:Basic printed versus usual care, lifetime	33E+07*	4,59E+08	44380*	9174	0,003
	Computer -tailored PA intervention:Web-based basic versus usual care, lifetime	9E+07*	1,25E+08	9150*	2502	

*cumulative lifetime

**Incremental QALYs estimated by for breast cancer alone (Bos 2019, personal communication) were above the minimum required for $50,000.
